# Application of ultra-weak photon emission imaging in plant stress assessment

**DOI:** 10.1007/s10265-024-01600-w

**Published:** 2025-01-05

**Authors:** Ankush Prasad, Eliška Mihačová, Renuka Ramalingam Manoharan, Pavel Pospíšil

**Affiliations:** Department of Biophysics, Faculty of Science, Palacký University, Šlechtitelů 27, Olomouc, 779 00 Czech Republic

**Keywords:** Antioxidants, Reactive oxygen species, Stress imaging, Two-dimensional photon emission imaging, Wounding

## Abstract

The oxidative damage induced by abiotic stress factors such as salinity, drought, extreme temperatures, heavy metals, pollution, and high irradiance has been studied in *Arabidopsis thaliana*. Ultra-weak photon emission (UPE) is presented as a signature reflecting the extent of the oxidation process and/or damage. It can be used to predict the physiological state and general health of plants. This study presents an overview of a potential research platform where the technique can be applied. The results presented can aid in providing invaluable information for developing strategies to mitigate abiotic stress in crops by improving plant breeding programs with a focus on enhancing tolerance. This study evaluates the applicability of charged couple device (CCD) imaging in evaluating plant stress and degree of damage and to discuss the advantages and limitations of the claimed non-invasive label-free tool.

## Introduction

Plants are exposed to various stresses in their life cycle, biotic stresses such as pathogen/contagion, herbivory, and abiotic stress factors such as salinity, drought, extreme temperatures, heavy metals, pollution, and high irradiance. All of the above stress factors lead to higher formation of reactive oxygen species (ROS) and reactive nitrogen species (RNS) predominantly due to disturbance of cell homeostasis (Das and Roychoudhury 2014). However, the survival of plants depends on many important factors, such as changes in growth conditions, severity and duration of stress conditions, and the ability to adapt quickly to changes in the environment (Miller et al. 2010).

**Fig. 1:**
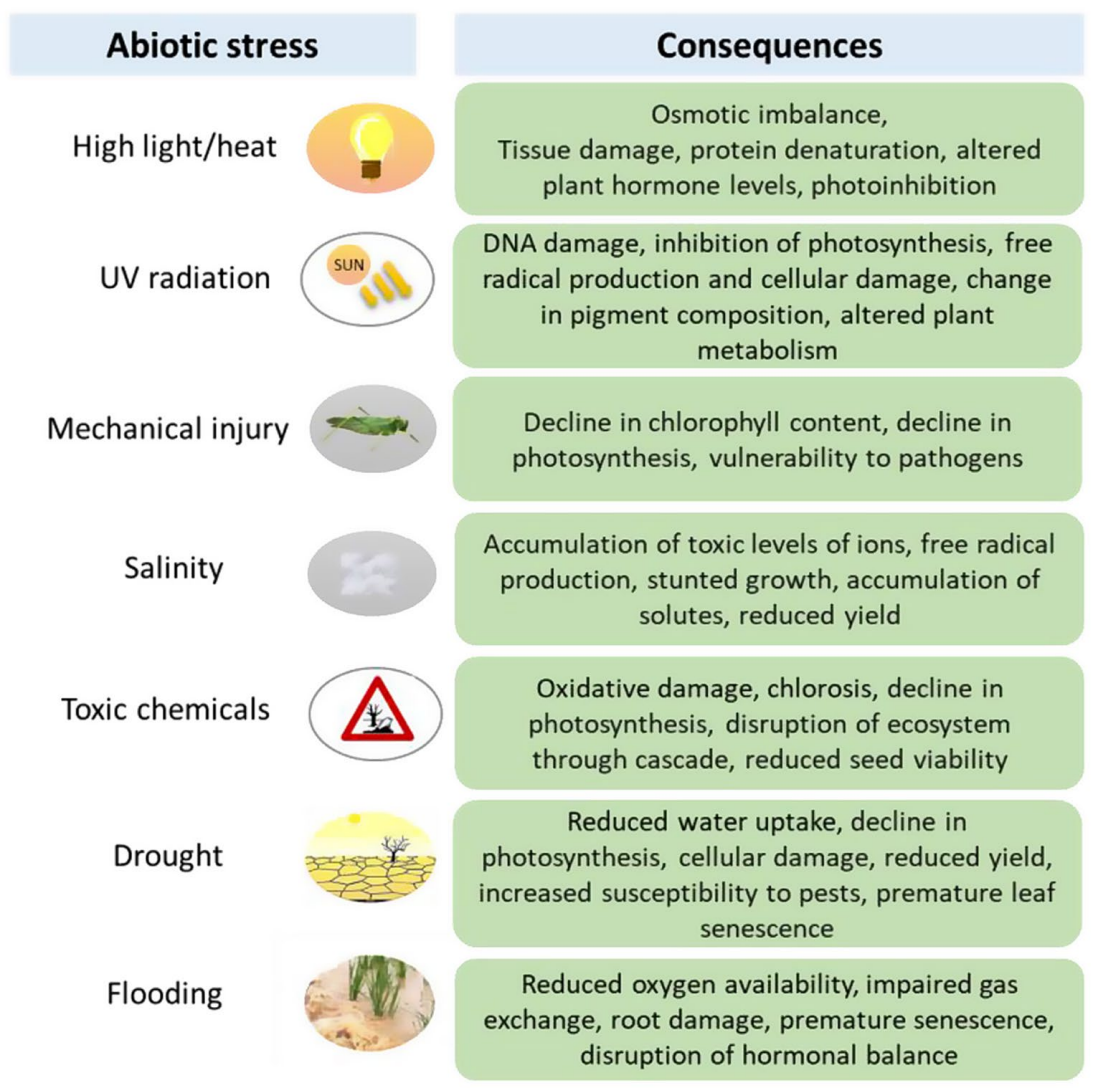
Abiotic stress on plants and its consequences

The lower concentration of ROS and RNS facilitates signals that induce important reactions in plant cells, such as controlling growth and development, and aiding in the development of tolerance to environmental stresses (Desikan et al. 2001; Neill et al. 2002; Yan et al. 2007). At high levels, however, they cause damage to cellular components that trigger oxidative stress (Dat et al. 2000; Mittler et al. 2004). Thus, whether ROS is destined to serve as signaling molecules or induce oxidative damage depends entirely on the delicate equilibrium between production and their scavenging by the antioxidant system present within the living organism. It has been known in plants that if stress is continuous, ROS accumulates in the leaves, resulting in the oxidation of many critical cellular components (proteins, lipids, nucleic acids), causing disruption of cellular metabolism that leads to cell death through the activation of the pathway involved (Apel and Hirt 2004; Foyer and Noctor 2005). Reactive oxygen species/RNS generation can cause the oxidation of biomolecules, which eventually, via the formation of reactive intermediates, can lead to the formation of species such as singlet oxygen, triplet carbonyls, etc. responsible for photon emission in the blue-green, red, and near-IR region of the spectrum (Burgos et al. 2017; Cifra and Pospíšil 2014; Gutteridge and Halliwell 2000). A summary of the involved mechanism is presented in Fig. 1, details of which have been previously described (Burgos et al. 2017; Cifra and Pospíšil 2014; Pospíšil et al. 2019).

Technology developed during the past decade has led to faster and more precise methods to quantify the effects and damages caused by oxidative stress. The development of a simple tool that can indicate the physiological state by reflecting the degree of oxidative stress would be an innovative step toward addressing this dynamic process in biological systems. There have been fewer studies on the application of ultra-weak photon emission imaging, and researchers around the world have applied it to different plant systems (Flor-Henry et al. 2004; Kato et al. 2014; Prasad et al. 2016, 2020a). In the current study, we performed experiments to check the applicability of charge-coupled device (CCD) imaging to evaluate plant stress and degree of damage in response to different abiotic stresses such as heat, salt, oxidant, mechanical wounding, and/or their combinations. Ultra-weak photon emission is proposed as a potential tool to measure oxidative processes due to its association with ROS/RNS-driven oxidative radical reaction (Pospíšil et al. 2014; Slawinski et al. 1992).

Ultra-weak photon emission is characterized as non-thermal radiation in the near-ultraviolet to the visible region (100–800 nm) of the electromagnetic spectrum, possibly reaching the near-infrared region (800–1300 nm). This phenomenon is considered a spontaneous aspect of any biological system, and has been experimentally shown to directly correlate with the cellular processes within the living system (Poplová et al. 2023). An increase in ultra-weak photon emission can be associated with higher metabolic processes and/or higher oxidative radical reactions, including those activated in response to abiotic stress (Floryszak-Wieczorek et al. 2011; Kato et al. 2014). Thus, ultra-weak photon emission is an impending new tool for monitoring dynamic biological processes and evaluating physiological /or pathological states in a living system (Burgos et al. 2016; Rastogi and Pospíšil 2011). A clear advantage of ultra-weak photon emission is that it provides spatiotemporal information (Kobayashi 2005; Usui et al. 2019). In addition, it is non-damaging, non-invasive, label-free, and relatively cost-effective (Cifra and Pospíšil 2014; Zhao et al. 2017). Photomultipliers (PMTs) can be used in conjugation with CCD cameras if ultra-weak photon emission kinetic is desired.

The CCD chip used in the current study was cooled by the liquid nitrogen system to decrease thermal noise and improve the S/N ratio (Faruqi 1998; Martin and Bronstein 1994). The quality of the image is determined by detector efficiency, that is, the fraction of incident photons absorbed in the detector; intensity (z-axis) and spatial resolution (x-y-axis), which depend on the pixel size (several units to tens of mm); ranges from 512 × 512 pixels up to 1800 × 1800 (Kobayashi et al. 2009; Prasad and Pospíšil 2011b, 2012; Rastogi and Pospíšil 2011) or more. Sensitivity limitations can be overcome by extending the charge accumulation time, in addition to applying binning, which combines adjacent pixel charges and offers benefits in faster reading speeds and improved S/N ratios, although at the expense of reduced spatial resolution (Kobayashi et al. 2009; Prasad and Pospíšil 2012). CCD technology was largely designed for application in astronomical studies, but the availability of a highly sensitive chip has made it possible to image two-dimensional ultra-weak photon emission (Kobayashi et al. 2009; Prasad and Pospíšil 2011b). Therefore, in our present study, it was used to visualize spontaneous photon emission and to evaluate the extent of oxidative damage induced in *Arabidopsis thaliana* (L.) by a different set of abiotic stressors. Ultra-weak photon emission is presented as a signature reflecting the extent of oxidative process and/or damage; to discuss the advantages and limitations of the claimed non-invasive label-free tool. The study presents an overview of a potential research platform where the technique can be applied. The results have been complemented with the use of different scavengers and inhibitors to address the involvement of ROS, and a kinetic study using a PMT was performed to validate the findings, which was further supported by monitoring the level of malondialdehyde (MDA) formation.

## Materials and methods

### Arabidopsis plant and growth conditions

The seeds of Arabidopsis WT (Columbia-0) were purchased from the Nottingham Arabidopsis Stock Centre at the University of Nottingham (Loughborough, UK). The seeds were first soaked in distilled water for the first 4 days at 4 °C and potted in growing pots filled with a peat substrate (Klasmann, Potground H). The plants were grown under the following conditions: photoperiod, 8/16 h light/dark; photon flux density, 100 µmol photons m^− 2^ s^− 1^; temperature, 22 ˚C/20 ˚C light/dark and relative humidity, 60% in Fytoscope FS-WI-HY (Photon Systems Instruments, Drásov, Czech Republic). Plants aged between 6 and 7 weeks were used for the study.

### Stress exposure and chemical reagents

Abiotic stresses were imposed, and their details are mentioned below. The chemicals used and their final concentrations are as follows: 5 mM H_2_O_2_ (Sigma-Aldrich, Darmstadt, Germany); 10 mM sodium chloride (NaCl) (PENTA Chemicals, Chrudim, Czech Republic). For heat stress, Arabidopsis was treated at high temperature (45 °C) for 30 min in a thermostat under dark conditions. After treatment, the plants were immediately transferred to the darkroom, and the ultra-weak photon emission was measured without any delay (the transfer time was kept to a minimum of about 30 s). For photooxidative stress, a photon flux density of 1000 µmol photons cm^− 2^ s^− 1^ was applied for 30 min on the whole plant. The light intensity was measured and adjusted using an LI-250 A light meter (LI-COR Biosciences, Lincoln, Nebraska USA). Surgical needles and micropipettes (using glass micropipettes, Blaubrand GmbH Germany) were used to induce wounding/mechanical injury to plant leaves. To study the combination of stresses, the same plants were used. Mechanical injuries were made by making a V-shaped cut or several holes in the leaves.

The application of salt stress was achieved by adding 1 ml of 10 mM NaCl to the peat substrate for 18 h. The preparation was done overnight before the day of experiments, and the addition of NaCl solution was done close to the roots on the peat substrate. Similar protocol was used for hydrogen peroxide (H_2_O_2_); however, the duration of exposure to the chemical was limited to 3 h before the measurement.

To confirm the role of ROS and oxidative processes in ultra-weak photon emission, we tested the effect of topical application of ascorbic acid (1 mM), histidine (10 mM) and superoxide dismutase (SOD) (400 U ml^− 1^) (Sigma-Aldrich Chemie GmbH, Germany) on the mechanically injured site.

### Charge-coupled device imaging

For the imaging of two-dimensional ultra-weak photon emission, we used the VersArray 1300B CCD camera (Princeton Instruments, Trenton, NJ, USA). The camera has a spectral sensitivity of 350 to 1000 nm and a quantum efficiency of about 90%. To improve light collection efficiency, a 50 mm objective lens (F Nikkor 50 mm, f: 1.2, Nikon) was used. Cooling the CCD camera to -110 °C using a liquid nitrogen cooling system effectively reduced noise. The following measurement parameters were used: a scan rate of 100 kHz, a gain of 2, and accumulation times of 30 min; image format of 1340 × 1300 pixels. To improve image quality and signal noise (S/N) ratio, background signal subtraction was performed before each measurement for data correction. The CCD camera was placed in a black box in an experimental dark room. The room was specifically designed with black walls and a door protected by a black curtain to avoid any potential interference by external light. The data-recording computer was located in an outer room, also without external strong interference of light. Before each measurement (except where photooxidative stress was applied), plants were transferred to the darkroom and were allowed to adapt for 2–3 h. This time duration is sufficient to completely get rid of any interference of absorbed/ delayed photons.

### Kinetics of ultra-weak photon emission

The kinetics of ultra-weak photon emission were performed using a photomultiplier tube (PMT) R7518P that has a spectral sensitivity between 185 and 730 nm. For the reduction of thermal electrons, PMT was cooled down to -30 °C using a thermoelectric cooler (C9143, Hamamatsu Photonics K.K., Iwata City, Japan). Measurements were made at 25 ℃ and the photon counts were recorded using a low-noise photon counting unit (C9744, Hamamatsu Photonics K.K., Iwata City, Japan). The PMT was mounted vertically from the top of a black box in an experimental dark room, avoiding any potential interference by external light. All other setup and experimental conditions remain unchanged from those outlined in Methodology mentioned above.

### SDS-PAGE and western blotting

To detect MDA-protein adduct formation as a result of abiotic stress, Arabidopsis leaves (0.5 g/ variant) were taken for each experimental condition described in Sect. 2.2. Protein extraction from the samples was carried out by freezing the samples in liquid nitrogen followed by crushing for 5 min in extraction buffer [SDS 12% w/v, β-mercaptoethanol 6% w/ v, glycerol 30% w/v and Tris/HCl (150 mM, pH 7.0)]. After crushing, the samples were filtered through two (2) layers of cheesecloth (in ice). The extract was then centrifuged at 16,000 rpm for 10 min and supernatants were incubated at 70 °C in the dry bath for 15 min followed by a second centrifugation for 10 min at room temperature. The supernatant was loaded into 10% SDS gel and using Mini-PROTEAN Tetra vertical electrophoresis cell, (Bio-Rad, CA, United States) electrophoresis was completed following the protocol described (Schagger 2006). Proteins resolved in SDS gels were transferred to a nitrocellulose (NC) membrane using a semi-dry blotter (Trans-Blot SD, Semi-dry transfer cell, Bio-Rad, United States). To detect MDA adducts formed on the proteins, the NC membrane was incubated for 3 h with 5% BSA prepared in phosphate-buffered saline-tween 20 (PBST; pH- 7.4) at 4 °C to prevent binding of antibodies to the non-specific binding sites on the NC membrane. All the following steps were performed on a shaker at room temperature. After blocking of non-specific binding sites on the NC membrane, it was incubated with rabbit polyclonal anti-MDA (1:5000, Abcam) for 90 min followed by three washes of 10 min each with PBST. The NC membrane was incubated with horseradish peroxidase (HRP) conjugated goat anti-rabbit secondary antibody (1:10000, Bio-Rad) for 1 h followed by washing as before. The bands were visualized using luminol as a chemiluminescent probe and images were captured by AI600 (Amersham Imager 600, GE Health Care Europe GmbH, Freiburg, Germany). For the identification of the size of bands, we used a standard protein ladder (ProteinTech prestained protein ladder, 10 to 180 kDa, PL00001).

## Results and discussion

### Spontaneous ultra-weak photon emission from Arabidopsis

**Fig. 2:**
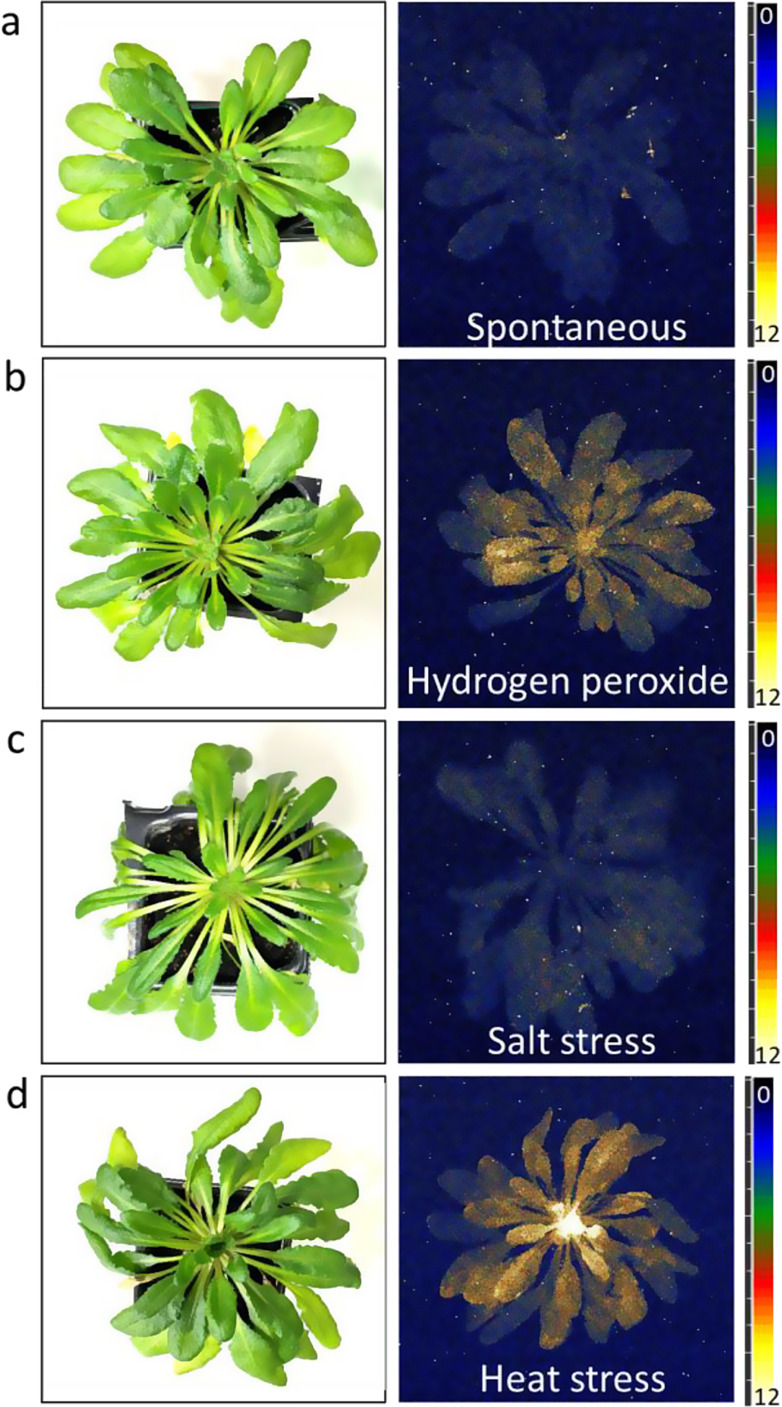
The photograph (left) and the two-dimensional image of ultra-weak photon emission (right) from Arabidopsis measured with an accumulation time of 30 min. In panel **a**, the right image shows spontaneous emission. In panel **b**, exogenous application of an oxidant (right image) was performed using 5 mM H₂O₂ for 3 h, applied directly to the peat substrate near the roots. In panel **c**, salt stress was induced using 10 mM sodium chloride for 18 h, and in panel **d**, heat stress was applied at 45 °C for 30 min in the dark

Figure 2a shows the photograph (left) and spontaneous ultra-weak photon emission (right) from Arabidopsis. Spontaneous ultra-weak photon emission refers to the photon emission occurring in living organisms because of metabolic oxidative reactions, for example, during photosynthesis or cellular respiration (Foyer 2018; Prasad and Pospíšil 2011a). It has been reported that the intensity can vary during the day, and usually, the strongest is at the forenoon (Flor-Henry et al. 2004). This photon emission is mostly associated with cellular activities, plant metabolism, and photosynthesis, and has been long known to be associated with various biochemical and physiological processes within plant cells/ tissues. It can be seen that the spatial distribution of photon emission is uniform throughout the leaf surface (Fig. 2, right panel). Based on the ultra-weak photon emission signature, the physiological state and the overall health can be predicted.

### Ultra-weak photon emission induced by hydrogen peroxide

Hydrogen peroxide is an oxidizing agent which in the presence of transition metal ions produces highly reactive hydroxyl radical (HO^•^) (Prousek 2007). The HO^•^ formed are capable of oxidizing organic molecules thereby leading to the formation of reactive intermediates or oxidation products. In this study, it was used to mimic endogenous ROS generated through photochemical reactions, enzymes, industrial wastes, or toxic chemicals that directly or indirectly reach the plants. Results from H_2_O_2_-treated Arabidopsis show enhanced intensity of photon emission (Fig. 2b, right panel). It can be seen that the overall ultra-weak photon emission is higher compared to spontaneous photon emission (Fig. 2a, right panel) however some spots within the sample are more intense which can refer to areas where higher damage to biomolecules occurred thereby leading to more emission. It can also indicate higher update/ diffusion of oxidant to these areas within the leaf tissue.

### Ultra-weak photon emission induced by salt stress

Salt stress in plants occurs when the concentration of salts (predominantly NaCl) in the soil or surrounding environment exceeds the tolerance level resulting from the usage of saline water for irrigation, poor irrigation drainage leading to accumulation of salt, and seawater intrusion, etc. Our measurement shows a marked difference in the intensity of ultra-weak photon emission between the control (Fig. 2a, right panel) and the salt-treated Arabidopsis (Fig. 2c, right panel). Higher intensity of ultra-weak photon emission is visible in Fig. 2c (salt-stressed plant) compared to non-treated plant (Fig. 2a). Excessive sodium accumulation in the cytoplasm impairs essential physiological processes such as nutrient uptake and electron transport. These disruptions can result in leakage of electrons in the electron transport chain and can lead to the formation of O_2_^•−^. It has also been reported that salt stress can activate Nicotinamide adenine dinucleotide phosphate hydrogen (NADPH) oxidase and studies have indicated towards the disruption of antioxidant defense systems (decreased activity or depletion of antioxidant enzymes such as SOD, catalase, peroxidases, and glutathione peroxidases).

### Ultra-weak photon emission induced by heat stress

Figure 2d shows the effect of heat treatment on the intensity of ultra-weak photon emission measured in Arabidopsis. The plants were treated at 45 ºC for 30 min and images were captured with an accumulation time of 30 min. A higher intensity of photon emission was observed, especially in parts located in the center, and comparatively lower intensity around the periphery of the plant. Broadly, the ultra-weak photon emission was significantly higher compared to the control (non-heat-treated sample) (Fig. 2a, d, right panels). When plants are subjected to heat stress, they are known to undergo a series of physiological and biochemical changes. It has been reported that changes in the excitation state of photosynthetic pigments, ROS production, and alteration in cellular metabolism can lead to the oxidation of biomolecules triggering ultra-weak photon emission via the formation of reactive intermediates and an electronically excited product (Allakhverdiev et al. 2008; Choudhury et al. 2017; Marutani et al. 2012; Pospíšil and Yamamoto 2017).

Understanding and interpreting the ultra-weak photon emission signatures from plants can have potential applications in agriculture and plant physiology. It can aid valuable information for developing strategies to mitigate heat stress in crops via improving plant breeding programs with a focus on enhancing heat tolerance. Additionally, ultra-weak photon emission can be used to assess the severity of heat, predict the plant responses, and optimize the cultivation procedures. However, it requires a larger industrial setting allowing scanning of more extensive surfaces.

### Ultra-weak photon emission induced by photooxidative stress or/and mechanical injury

**Fig. 3:**
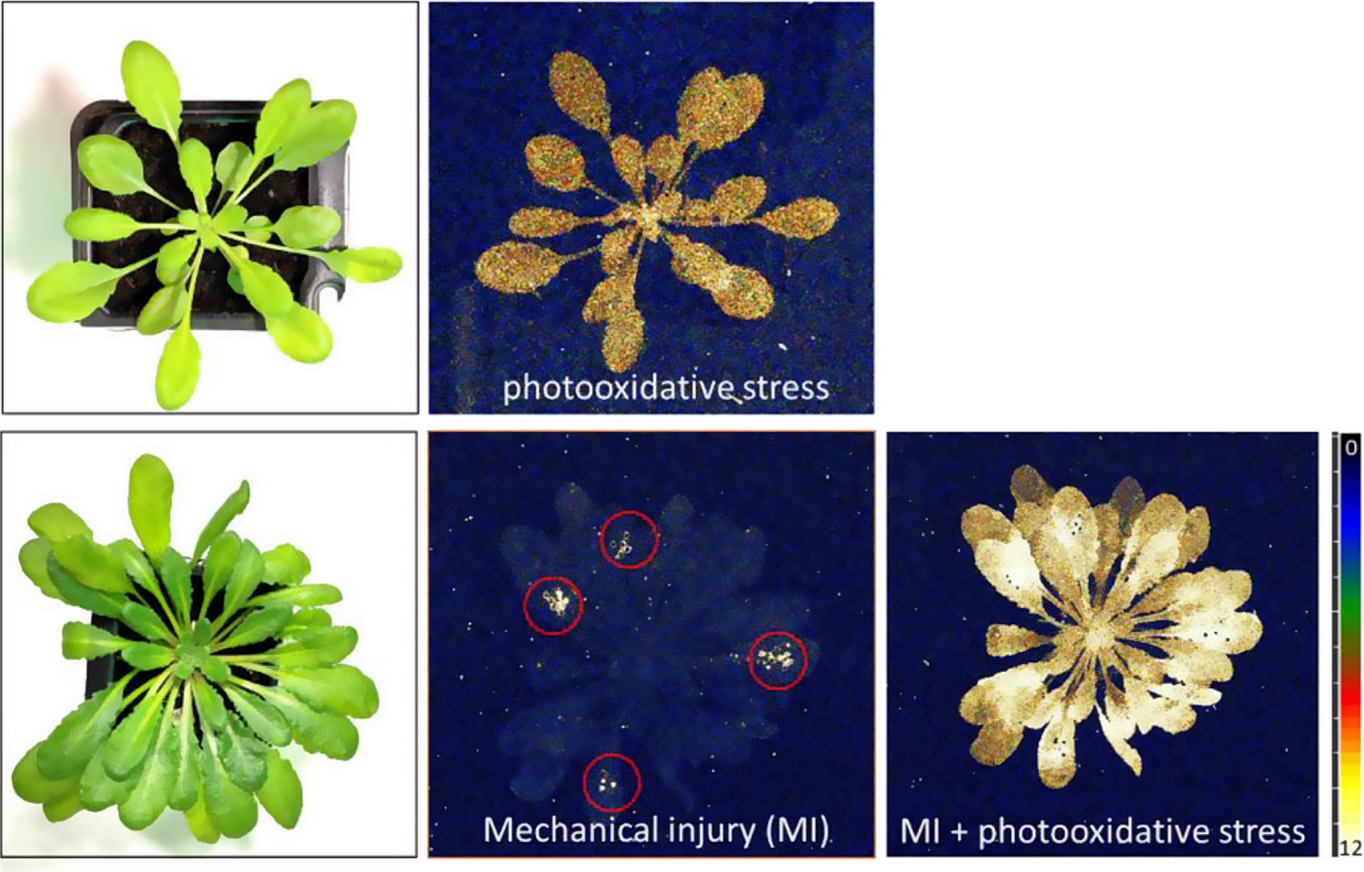
Photograph (left) and two-dimensional imaging of ultra-weak photon emission from photooxidatively stressed Arabidopsis (upper panel) and photon emission induced by mechanical injury and in combination with photooxidative stress (lower panel)

Figure 3 shows the photographs (left) and the influence of mechanical wounding either in the absence (middle) or presence (right) of photooxidative stress in Arabidopsis. In the middle panel, the plant was mechanically wounded, while in the right panel, it was exposed to photooxidative stress (1000 µmol photons cm^− 2^ s^− 1^) for 30 min. As a control, the upper panel illustrates the effect of photooxidative stress alone.

The mechanical injury was performed in the presence of diffused green light followed by a dark adaptation again for 10 min. The time duration was chosen on kinetic measurement using a photomultiplier tube (PMT); it was assured that there is no more delayed luminescence due to green light exposure (data not shown). After measurement, the mechanically injured plant was further exposed to high light to understand whether a combination of stress has any effect on the mechanical wounded site. At most of the mechanically injured parts of the leaves, it can be observed that the intensity of ultra-weak photon emission is highest compared to other parts of the plant leaves. Additionally, it can also be seen that the older leaves with high chlorophyll content seemingly show higher intensity of photon intensity. Similar observations were perceived in previous studies where mature leaves exhibited higher photon emission than young leaves located in the center of the plant (Havaux et al. 2000, 2009). When plants experience mechanical wounding, they undergo a series of responses at cellular and molecular levels (Prasad et al. 2020b; Savatin et al. 2014). Damaged tissues undergo rapid cell division and produce callus tissues; plant hormones such as jasmonic acid, ethylene, and salicylic acid are produced and redistributed, which then regulates signaling pathways to trigger defense responses; activation of defense genes; induction of proteinase inhibitors and formation of lignin and further strengthening of cell walls (Leon et al. 2001; Orozco-Cardenas et al. 2001; Prasad et al. 2020a, b; Rehrig et al. 2014; Zhao et al. 2010). In addition to the above responses, the cells also produce ROS which contributes to its crucial role in the activation of signaling pathways and triggering various defense responses. It has been reported that activation of enzymes such as NADPH oxidase and peroxidases produce O_2_^•−^ and H_2_O_2_, respectively. Mechanical injury triggers the influx of calcium ions (Ca^2+^) towards the wounded site which acts as a secondary messenger for stimulating ROS production (Beneloujaephajri et al. 2013; Orozco-Cardenas et al. 2001; Prasad et al. 2020b). Our presented results can show the extent of these mentioned processes at the injury and in conjugation with other methodologies can help understand the mechanisms involved.

### ROS, oxidative damage, and ultra-weak photon emission

**Fig. 4:**
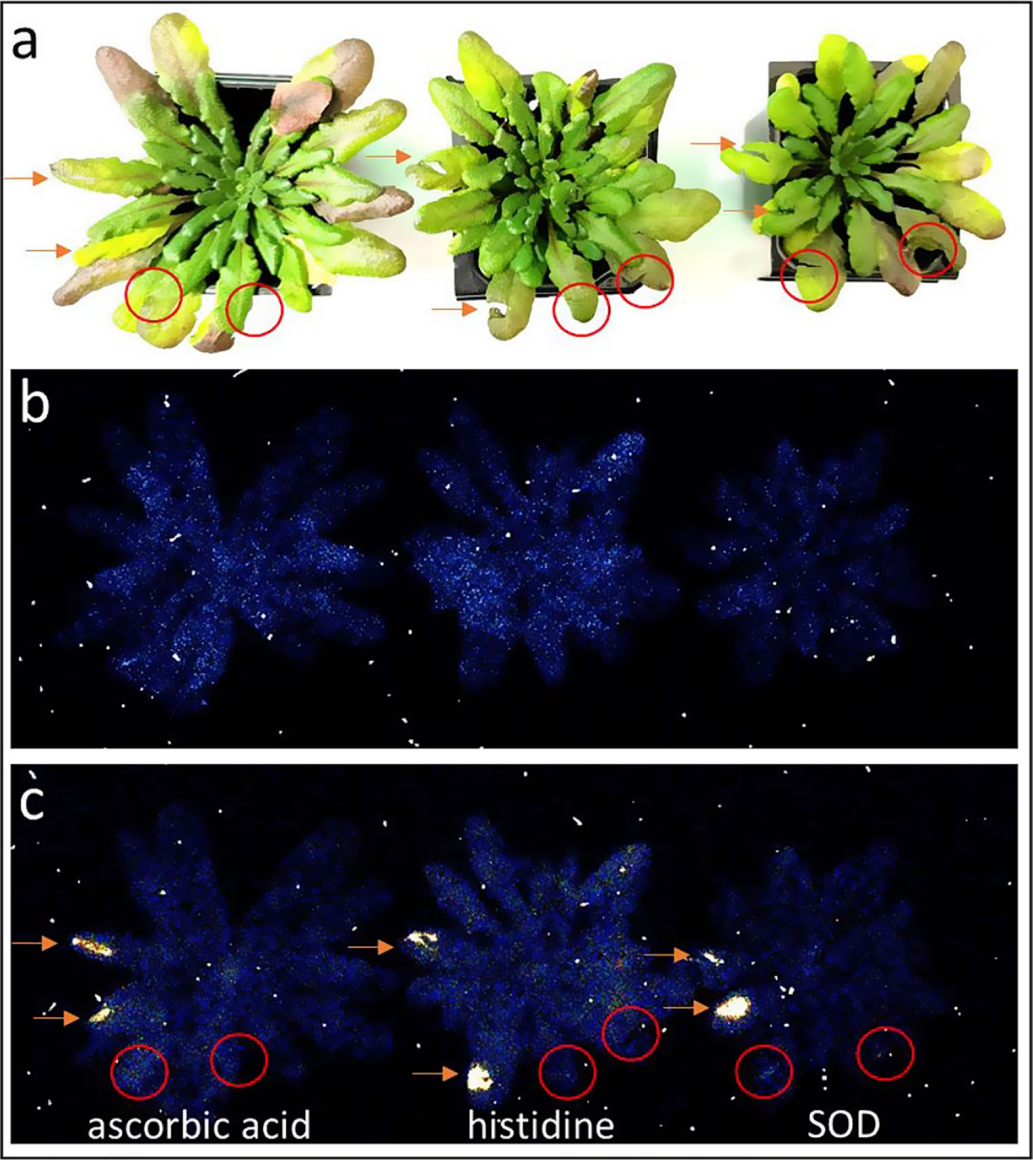
Photographs (**a**) and two-dimensional images of spontaneous Arabidopsis plants (**b**) and mechanically injured (**c**). In panel B, no mechanical injury was made, and two-dimensional imaging of spontaneous ultra-weak photon emission was measured as in Fig. 2a. In panel **c**, mechanical injury was done on four different leaves marked with red arrows and circles. In panel **c**, ascorbic acid, histidine, and SOD were applied immediately after injury in the leaves marked with a circle and the arrows indicate the untreated wounded leaves of the same plant

**Fig. 5:**
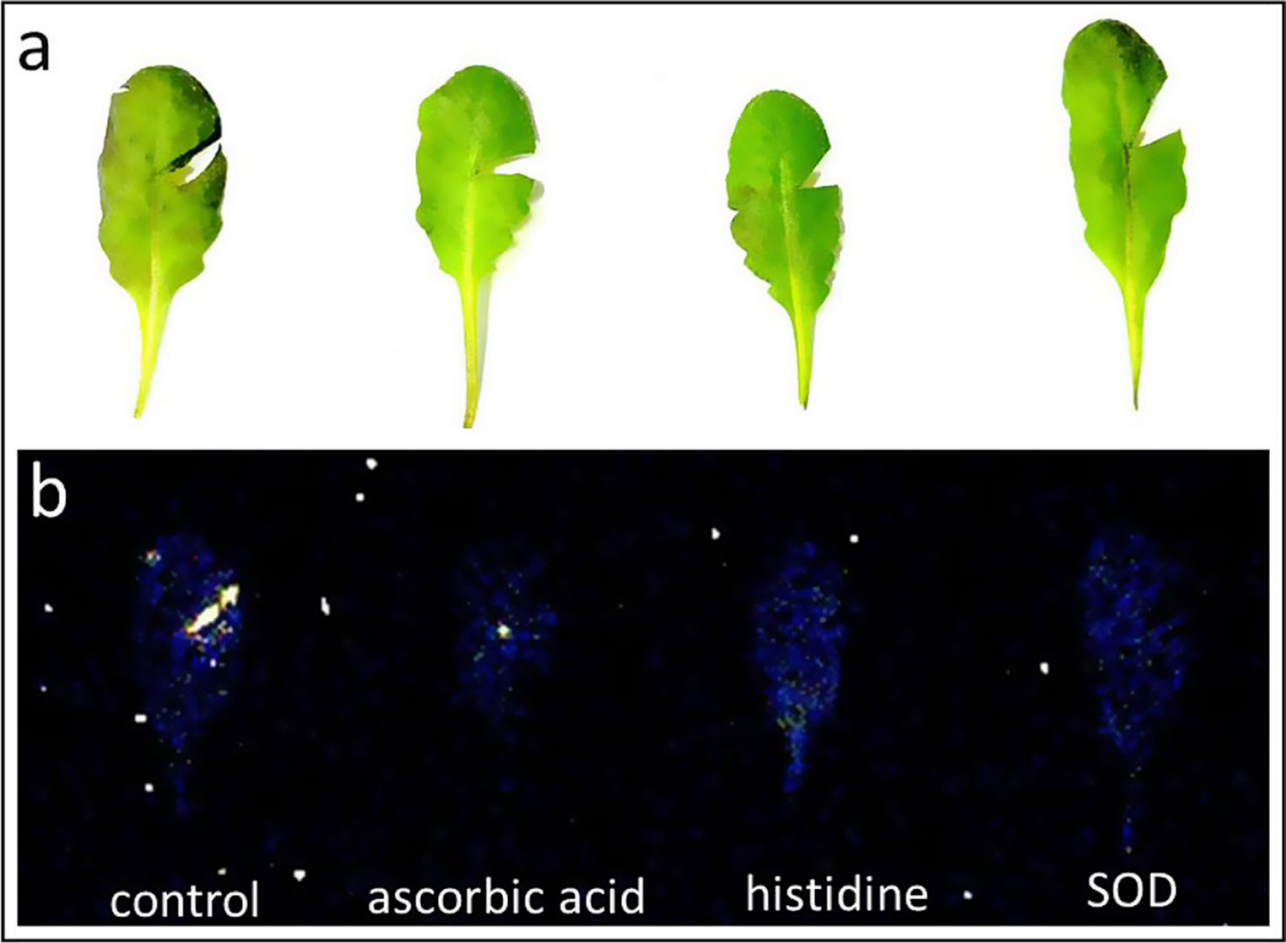
Photographs (**a**) and two-dimensional images of mechanically injured Arabidopsis leaves. In panel **b** (control), mechanical injury was made while on other leaves, immediately following the mechanical injury, either of the compounds as mentioned in the panel were applied and two-dimensional imaging of ultra-weak photon emission was measured

Abiotic stress in plants is recognized to induce ROS production, ultimately resulting in an increase in ultra-weak photon emission through oxidative radical reactions, the formation of electronically excited species, and the generation of a final emitter, as detailed also in our recent reviews (Poplová et al. 2023; Pospíšil et al. 2019). To substantiate these claims, we performed measurements of ultra-weak photon emission in mechanically injured plants (Fig. 4) and leaves (Fig. 5), both in the absence and presence of inhibitors and ROS scavengers. Mechanical injury provided a very marked difference at the site of injury compared to spontaneous emission from neighboring tissue, making it the optimal selection to assess the effect. Therefore, we chose ascorbic acid, histidine, and SOD and applied them on mechanically injured Arabidopsis plants and detached leaves. It can be clearly seen that at the site of topical application of these compounds, the photon emission intensity is suppressed either partially or even completely. In the case of ascorbic acid, there appears to be partial suppression in the case of leaves (Fig. 5b), while in the case of the Arabidopsis plant, there is almost complete suppression of ultra-weak photon emission. In the case of histidine and SOD, complete suppression can be observed similar to our previous reports (Prasad et al. 2020b). Ascorbic acid works as a scavenger of ROS and acts as an electron donor in addition to its chelating characteristics via binding to metal ions such as iron and copper. Histidine has been acknowledged for its ability to scavenge HO^•^ and singlet oxygen. L-histidine has been reported to interfere with redox reactions involving metal ions that eventually produce HO^•^ or by direct interactions of its histidine imidazole ring with singlet oxygen (Dogra and Kim 2020; Michaeli and Feitelson 1994; Wade and Tucker 1998). SOD being an essential enzyme in the body, plays a crucial role by catalyzing the dismutation of superoxide anion radical (O_2_^•−^) to H_2_O_2_. The process is vital in preventing the accumulation of O_2_^•−^ by converting them to less reactive species. As is also evident from the result and our previous report (Prasad et al. 2020b), there is apparently suppression in all cases, which brings us to the conclusion that these scavengers can rather work in different ways and target at different mechanistic pathways.

### Data validation using photomultiplier tube and western blotting

**Fig. 6:**
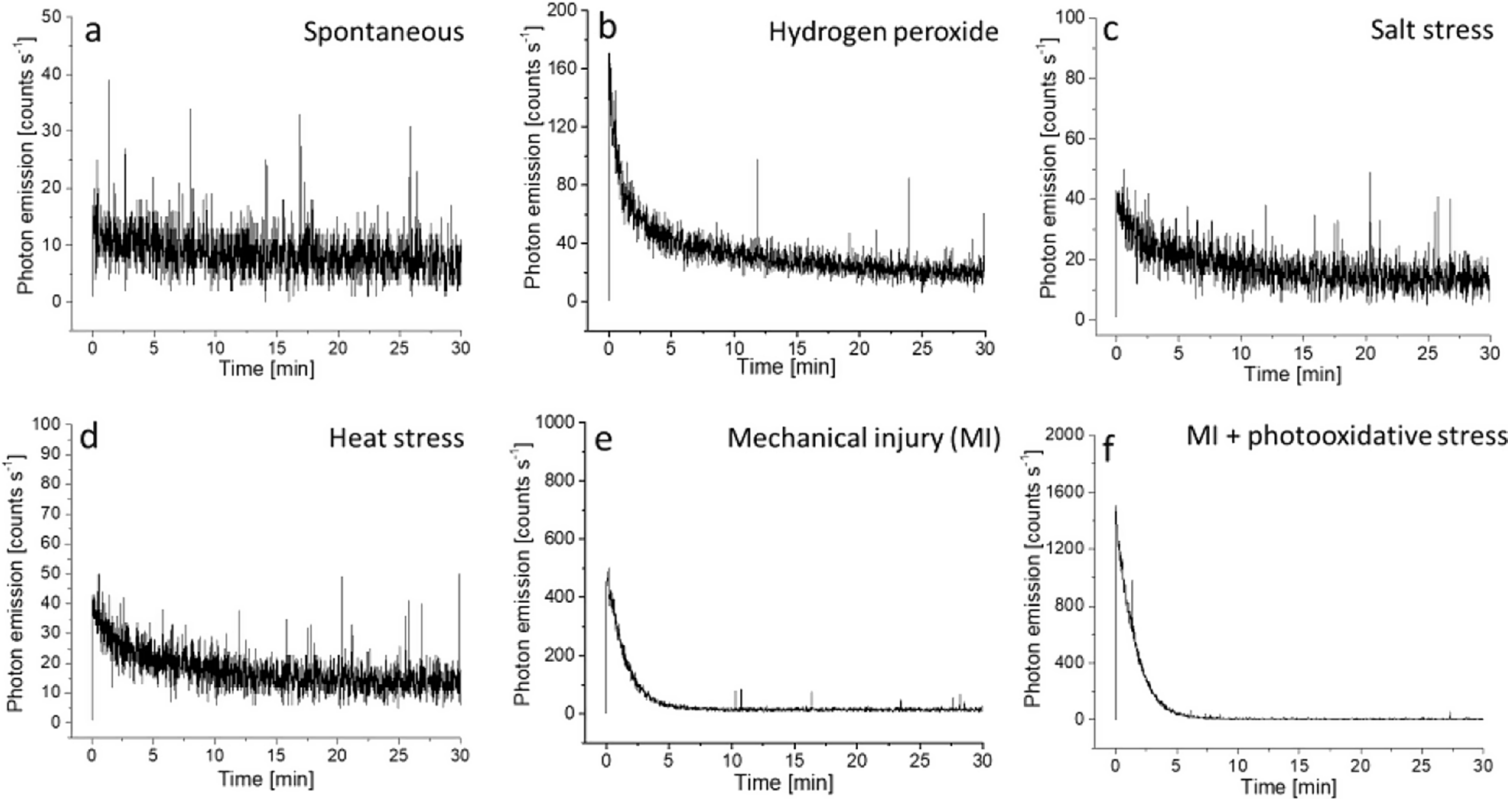
Abiotic stress-induced ultra-weak photon emission measured using visible PMT from Arabidopsis. The kinetics of ultra-weak photon emission was measured after exposure of Arabidopsis to H_2_O_2_, salt, heat, and mechanical injury with or without photooxidative stress. The doses and duration of treatment are as described in Sect. 2.2. The decay curve was measured for a duration of 30 min

**Fig. 7:**
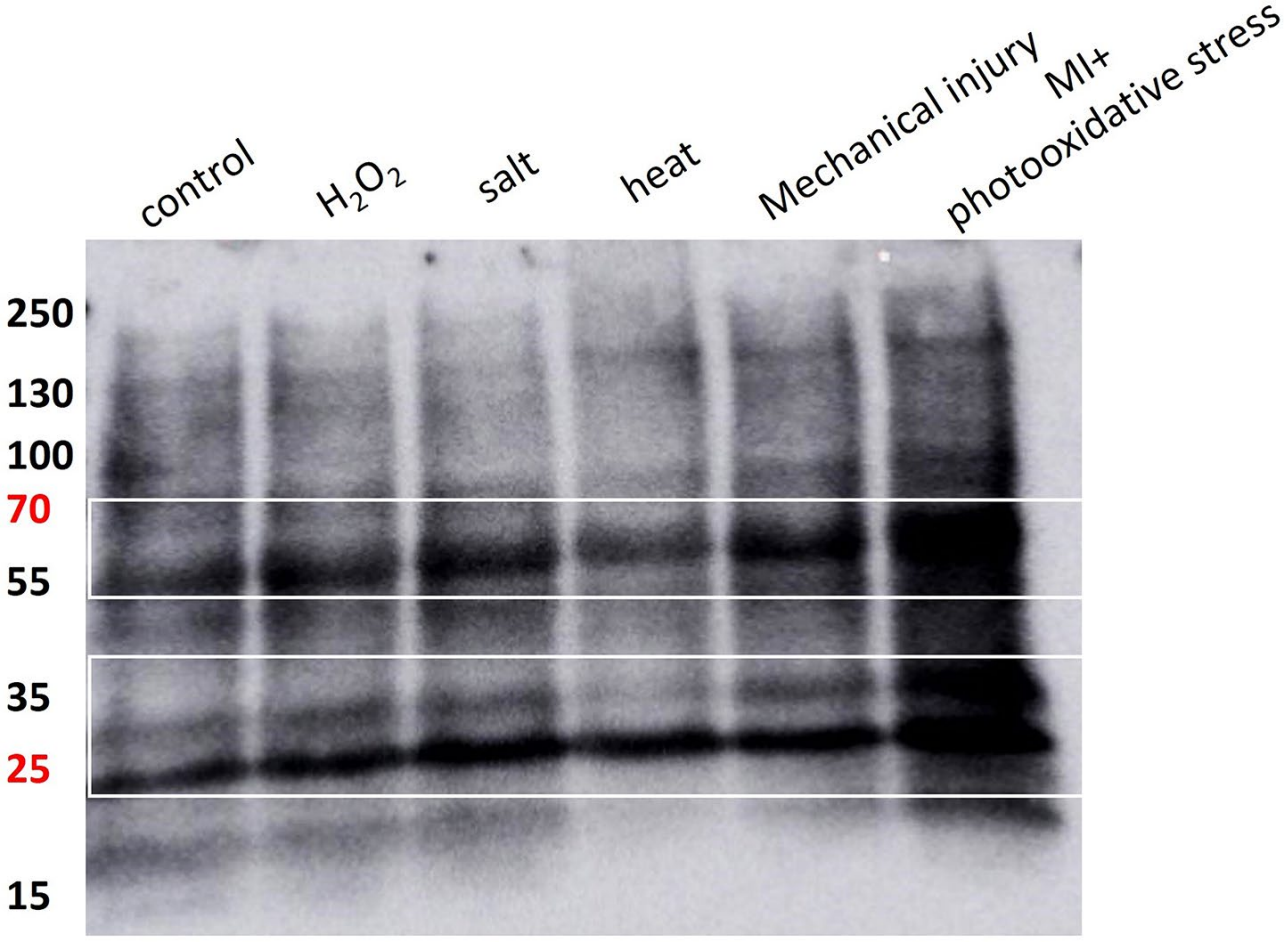
Detection of MDA-protein adducts in Arabidopsis control and abiotic stress conditions. MDA-protein adducts were detected with immunoblotting by conventional immunoblot techniques using anti-MDA antibody followed by HRP conjugated secondary antibody and luminol chemiluminescence on nitrocellulose membrane

To understand and validate the results obtained on two-dimensional imaging, we measured the kinetic behaviour of induced ultra-weak photon emission under the above abiotic stress. The kinetics of ultra-weak photon emission were studied following the same treatment condition as described in Section “Materials and Methods” with the exception that in case of salt stress, a reapplication of 1 ml of 10 mM NaCl was performed 1 h before measurement to observe detectable change in photon counts. When the Arabidopsis plant was subjected to H_2_O_2_, it can be observed that the ultra-weak photon emission was enhanced to ∼170 counts s^− 1^, which then decayed over time (Fig. 6b). When salt or heat stress was applied, the photon emission was not as pronounced as H_2_O_2_ with an intensity maximum of ∼40 counts s^− 1^ (Fig. 6c, d). Consistent with the imaging study, the highest photon emission was observed in the case of mechanical injury and mechanical injury in combination with photooxidative stress where the maximum photon intensity was recorded to be ∼500 counts s^− 1^ and ∼1500 counts s^− 1^ (Fig. 6e, f). The results were further confirmed through the assessment of MDA- protein adduct formation, and the corresponding findings are presented in Fig. 7. We measured the formation of MDA- protein adduct, which is a key marker of oxidative consequences. We employed protein immunoblotting, using an anti-MDA antibody, to examine protein modifications in both control and abiotic stress treated Arabidopsis leaves. Malondialdehyde is a reactive aldehyde that is formed primarily as a byproduct of lipid peroxidation. It can interact with proteins, primarily due to its reactivity as an aldehyde. These interactions can lead to modifications in protein structure and function, which contribute to cellular damage. In the blot presented in Fig. 7, it can be seen that the formation of MDA-protein adducts is evident at several positions (indicated by white boxes) within the blots and is more prevalent in abiotic stress corresponding to mechanical injury and MI + photooxidative stress (Fig. 7).

## Conclusion

Ultra-weak photon emission imaging, also referred to as biophoton imaging, is a unique technique used to study the photon emission of extremely low-level light from living organisms, including plants. This phenomenon is based on the generation of chemiluminescent signals which are ultra-weak in nature and can originate from various biological processes within the organisms. In our study, we have presented spatiotemporal images using a CCD camera that can be utilized to investigate how plants respond to various environmental stressors, such as light stress, temperature fluctuations, and pathogen attacks leading to mechanical wounding, etc. By monitoring changes in the intensity of photon emission, researchers can gain insight into the stress adaptation and defense mechanisms of plants. Until now, ultra-weak photon emission has primarily been employed to assess the extent of oxidative stress generated within a system, directly linked to cellular damage in cells or organisms. Plants recover from damage following mild or moderate stress, which is also our case. By tracking the signature of ultra-weak photon emission and delving into the intricacies of the recovery phase in plants, we are confident that there is considerable potential for expanding the application of this methodology. As technology develops and our understanding of plant biology expands, ultra-weak photon emission imaging may become an even more valuable tool for investigating plants’ responses to their environment and their internal physiological processes. The non-invasive nature of CCD imaging allows for real-time monitoring of photon emission, offering valuable insights into oxidative stress, cellular redox states, and other physiological processes in various biological samples. However, to obtain reliable results, key factors include conducting measurements in a dark environment, ensuring proper dark adaptation of samples beforehand, minimizing thermal noise, and performing accurate calibration through background subtraction should be considered.

## Data Availability

All data generated are included in either the manuscript or the supplementary dataset.

## Abbreviations

ROS: Reactive oxygen species
RNS: Reactive nitrogen species
CCD: Charge-coupled device
UPE: Ultra-weak photon emission
SOD: Superoxide dismutase
